# Supplementary material for: Chromothripsis during telomere crisis is independent of NHEJ, and consistent with a replicative origin
Source: Genome Res. 2019 May;29(5):737–49. doi: 10.1101/gr.240705.118 (PMC6499312; doi:10.1101/gr.240705.118)
Supplement: Supplemental Material [file supp_gr.240705.118_Supplemental_file_1.zip › contigs/annotated_contigs/DB113/contig.4.DB113_length_911_mean_cov_17.4533479693.docx]

**DB113_length_911_mean_cov_17.4533479693**

```
AGTGAGGCACCCAGCAAGGCCCCCAAGGGAAAAGAGGAAAGGAAGACCCCCTACACAGCCAGTCTGCTAGGAGAGGCCCTGGGCTTCTC
 >chr1:110793337-110793529 + E=1e-103
TAGGAGGAGGAGGAGAGGAGCGGCTGGGGTAGGGGAGGCTAGGCCAAGCCCATCAGGTGGAGAAGCAGGTGGCTGTTCCTGAAGCCTCA

AGCCCATGG|ACTGC|ATGCTAGTCCGGCTTGGCTTAAAGAGTCCACTTAGAGGACTTCAAGCTCCACAGGCCACGAACGTTGTTATGG
          >chr1:110793776-110793905 + E=1e-65
TTTGGCTGTGTCCCTACTCAAATCTCATCTTGAATTGTAGCACCCATA|ATT|TTTTTCAGTGACATTAAGTACATTCACATATTGTGC
                                                 >chr1:110792400-110792723 + E=2e-179
AACCTATATAGCCATCCATCTCCAGAACTCTTTCTGTCTTCCCAAACTGAGACTCTGCACCCATTAAACAATGACTCCTCATTCTCTTC

TCCCCCAGTCCCTAGCAACCAGCATTCTACTGTCTTTGTGAATTTGACTAAGTACCCCATATAAGTGGAATCATACGTTTTCAAGTCCC

ACCTTGAAGCTGTTATCAGCGAACACTTTCCTTAATTAAAAATTAACACCTTAGCAAGAATTAGTCTCTGACAACAGGAATGGGTCATC

ACTCCTTTTCTCATG|CC|CATAATTCCCATGTGTTGTGGGAAGACCTGGTGGGAAGTAATTGAATCATGGGGGCAGGTCTTTCCCATG
                >chr1:110793896-110794173 + E=1e-154
CTGTTCTCACAATAGTGAATACATCTCATGAGAAATGATGGTTTTATAAAGGGGAGTTCCCCTGCACACTCTCTCTTGCCTGCCGCCAT

GTAAGACATGTCTTTGCTCCTCCTTCACCTTTTGCCATGATTGTGAGGCACCCCCAGCCATGTGGAACTGTGAGTCCATTAAACCTCTT

TCTTTTGTAAATAACTCAGTCTCAGAT
```
